# A Novel Function for Fragile X Mental Retardation Protein in Translational Activation

**DOI:** 10.1371/journal.pbio.1000016

**Published:** 2009-01-20

**Authors:** Elias G Bechara, Marie Cecile Didiot, Mireille Melko, Laetitia Davidovic, Mounia Bensaid, Patrick Martin, Marie Castets, Philippe Pognonec, Edouard W Khandjian, Hervé Moine, Barbara Bardoni

**Affiliations:** 1 Institut de Pharmacologie Moléculaire et Cellulaire, Valbonne, France; 2 CNRS, UMR6097, Valbonne, France; 3 Université de Nice Sophia-Antipolis, Nice, France; 4 Institut de Génétique et de Biologie Moléculaire et Cellulaire, Illkirch, France; 5 CNRS, UMR 7104, Illkirch, France; 6 INSERM, U596, Illkirch, France; 7 Université Louis Pasteur 1, Strasbourg, France; 8 CNRS, FRE3094, Nice, France; 9 Neurobiologie Cellulaire, Centre de Recherche Robert Giffard, Université Laval, Québec, Canada; University of Wisconsin, United States of America

## Abstract

Fragile X syndrome, the most frequent form of inherited mental retardation, is due to the absence of Fragile X Mental Retardation Protein (FMRP), an RNA-binding protein involved in several steps of RNA metabolism. To date, two RNA motifs have been found to mediate FMRP/RNA interaction, the G-quartet and the “kissing complex,” which both induce translational repression in the presence of FMRP. We show here a new role for FMRP as a positive modulator of translation. FMRP specifically binds *Superoxide Dismutase 1* (*Sod1*) mRNA with high affinity through a novel RNA motif, SoSLIP (*Sod1* mRNA Stem Loops Interacting with FMRP), which is folded as three independent stem-loop structures. FMRP induces a structural modification of the SoSLIP motif upon its interaction with it. SoSLIP also behaves as a translational activator whose action is potentiated by the interaction with FMRP. The absence of FMRP results in decreased expression of *Sod1.* Because it has been observed that brain metabolism of *FMR1* null mice is more sensitive to oxidative stress, we propose that the deregulation of *Sod1* expression may be at the basis of several traits of the physiopathology of the Fragile X syndrome, such as anxiety, sleep troubles, and autism.

## Author Summary

The most common form of inherited mental retardation, Fragile X syndrome, is caused by the absence of the Fragile X Mental Retardation Protein (FMRP), which can bind mRNA. In mice devoid of FMRP, several hundreds of mRNAs with altered expression and localization have been reported. The impact of these abnormalities is particularly important in the brain, where the absence of FMRP affects the structure and function of connections between neurons, resulting in reduced cognitive abilities. In recent years, scientists studying Fragile X syndrome have focused on identifying the RNAs that are specifically bound by FMRP. We and others have identified two specific RNA structures bound by FMRP/RNA. Importantly, when FMRP binds these structures, the translation of the RNA into its protein product is inhibited. In this new study we have identified a third FMRP-binding RNA structure that is found in the mRNA that encodes superoxide dismutase, an oxidative-stress-mitigating enzyme. Most significantly, FMRP enhances translation of the superoxide dismutase mRNA when it interacts with the structure. These findings suggest that absence of FMRP might result in reduced levels of superoxide dismutase, which in turn leads to increased oxidative stress in the brain. Interestingly, oxidative stress in the brain has already been linked to anxiety, sleeping difficulties, and autism, all of which typically affect individuals with Fragile X syndrome.

## Abstract 3

Fragile X Mental Retardation Protein (FMRP), an RNA-binding protein mostly involved in translational repression, may also act as a translational activator of*Sod1* mRNA, suggesting that increased oxidative stress may be involved in the pathophysiology of Fragile X syndrome..

## Introduction

Fragile X Mental Retardation Protein (FMRP) is an RNA-binding protein whose absence causes the Fragile X syndrome, the most frequent form of inherited mental retardation [1]. An increasing body of evidence suggests that FMRP has a complex function, reflecting its involvement in the control of hundreds of mRNA targets via its different RNA-binding domains. Indeed, FMRP contains two heterogeneous nuclear ribonucleoprotein (hnRNP) K-homology (KH) domains and one arginine-glycine-glycine domain (RGG box) that can mediate protein/RNA interaction [1]. Although specificity of binding for the KH1 domain was not proved, the KH2 domain was shown to specifically bind a category of synthetic aptamers (“kissing complex”), a sequence-specific element within a complex tertiary structure stabilized by Mg^2+^ [2]. However, the RGG box is able to bind G-quartet RNA with high affinity [3,4]. This structure is present in several FMRP mRNA targets, such as *Fragile X Mental Retardation 1* (*FMR1*), *Microtubules Associated Protein 1B* (*MAP1B*), and *PP2Ac Protein Phosphatase 2A catalytic subunit* (PP2Ac) [3–5]. FMRP is able to shuttle between nucleus and cytoplasm, where it is mostly associated with polyribosomes, suggesting an implication in translational control [1]. In neurons, FMRP is also involved in RNA trafficking along dendrites and axons, being a component of RNA granules and functioning as a molecular adaptor between these complexes and the neurospecific KIF3C kinesin [6,7]. Moreover, after traveling along dendrites, FMRP associates with polyribosomes localized at the synapse to participate in the translational control of proteins synthesized in this compartment [8].

Taking into consideration the results obtained from different laboratories, several mechanisms of action of FMRP have been proposed suggesting: (i) polysomal stalling for *MAP1B* mRNA expression regulation [9]; (ii) retention of mRNAs in translationally inactive messenger RNPs (mRNPs) via its interaction with the kissing complex motif [2]; (iii) inhibition of translation preventing ribosome scanning via a G-quartet structure localized in the 5′ UTR of a target mRNA, as for the *PP2Ac* mRNA [5]. Moreover, the ability of FMRP to stabilize the *Post Synaptic Density 95* (*PSD95*) mRNA, by interacting with its 3′ UTR, was recently reported [10]. Here we show that FMRP interacts with the *Superoxide Dismutase 1* (*Sod1*) mRNA with high specificity and affinity via a novel RNA structure that we named SoSLIP (*Sod1* Stem Loop Interacting with FMRP). SoSLIP is organized in a triple stem-loop structure and acts as an FMRP-dependent translational enhancer and as a mild internal ribosome binding site (IRES) in an FMRP-independent manner. The characterization of this novel RNA motif interacting with FMRP sheds new light on the ability of this protein to bind RNA and to improve the translation of SoSLIP-containing mRNAs. Our results, taken together with other recent findings [11,12], suggest that the deregulation of *Sod1* expression may have an important role in the pathogenetic mechanism of Fragile X syndrome.

## Results

### FMRP Binds *Sod1* mRNA with High Affinity via Its C-Terminal Domain

**Figure 1:**
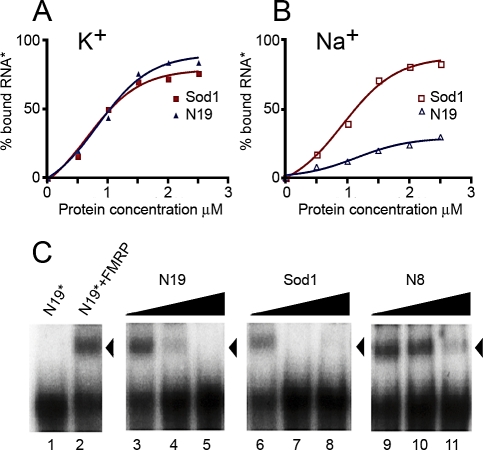
FMRP Specifically Binds *Sod1* mRNA FMRP binding to *Sod1* mRNA is not dependent on K^+^. Labeled G-quartet RNA (N19) or *Sod1* full-length mRNA were incubated with increasing amounts of recombinant His-FMRP in the presence of K^+^(A) or Na^+^(B). FMRP/*Sod1* binding was not affected by ionic conditions, whereas, as expected, the presence of Na^+^ affected FMRP binding to N19. (C) Gel-shift experiments were performed using a ^32^P-labeled N19 probe incubated with 0.1 pmol of recombinant His-tagged FMRP in the presence of increasing amounts of unlabeled competitors, ranging from 10^−9^ to 10^−7^M [lanes 3–5 (N19), lanes 6–8 (*Sod1*), lanes 9–11 (N8)]. Lane 1, no protein control; lane 2, no competitor control. Note that both N19 (positive control) and *Sod1* compete equally well for binding to FMRP, whereas N8 (negative control) only competes at high concentrations (nonspecific binding). All data obtained in these experiments are listed in Table S2.

**Figure 2:**
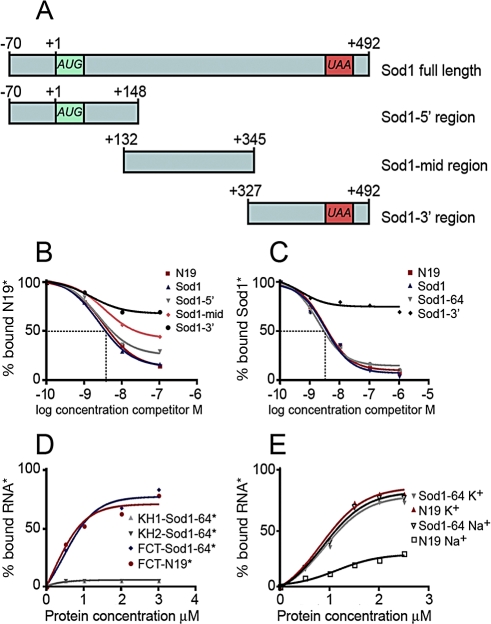
FMRP Binds a 64 Base Fragment of *Sod1* mRNA via Its C-Terminal Region (A) Schematic representation of *Sod1* mRNA and its fragments subcloned from full-length cDNA and used to map the binding domain of FMRP on *Sod1* mRNA. (B) Binding specificity of FMRP to *Sod1–5′* region. Filter binding assay using FMRP and ^32^P-labeled N19. The competition was performed using various regions of unlabeled *Sod1* mRNA: *Sod1–5′* region, *Sod1-mid* region, *Sod1–3′* region, and N19 itself. The graph shows the fraction of bound labeled N19 RNA plotted versus unlabeled competitor RNA concentration. (C) Binding specificity of FMRP to *Sod1–64* fragment. Filter binding assay using FMRP and ^32^P-labeled *Sod1* mRNA. Competition was performed with different unlabeled mRNA fragments, as indicated in the figure The *Sod1–64* RNA fragment shows a competition profile similar to that of *Sod1* full-length mRNA. (D) Filter binding assays using various recombinant RNA-binding domains of FMRP. KH1, KH2, and the C-terminal domain containing the RGG box and ^32^P-labeled RNAs reveal that the FMRP C-terminal domain displays equal affinity for *Sod1* mRNA or G-quartet (N19 fragment), whereas the two KH domains are not able to bind *Sod1* mRNA. (E) Filter binding assay using increasing amounts of recombinant His-FMRP and ^32^P-labeled RNA fragments in the presence of K^+^ or Na^+^. FMRP/*Sod1–64* RNA binding is not dependent on ionic conditions, excluding the presence of a G-quartet-forming structure RNA. All data are listed in Table S2.

With the goal to find novel mRNA structures specifically recognized by FMRP, we performed a systematic analysis of known FMRP mRNA targets, focusing on those that have been shown to interact in vivo with FMRP by the antibody-positioned RNA amplification (APRA) technique [13]. First, we excluded the presence of already known structures bound by FMRP in these mRNA targets by screening their capacity to bind a recombinant FMRP in the presence of Na^+^, K^+^, or Mg^2+^. Indeed, K^+^ ions stabilize the G-quartet RNA structure, leading to a robust interaction with FMRP [4], whereas Mg^2+^ favors FMRP/kissing complex RNA interaction [2]. This analysis resulted in the characterization of FMRP/*Sod1* interaction, which takes place in the presence of K^+^ (Figure 1A) and is not affected by the presence of Na^+^ (Figure 1B), whereas, as expected, Na^+^ affects the binding of FMRP to the N19 sequence that contains the G-quartet present in *FMR1* mRNA (nucleotide (nt) 1470–1496) [4]. Moreover, to definitely exclude the presence of a G-quartet structure in *Sod1* mRNA, we performed a reverse transcriptase (RT) elongation reaction assay, as previously described [4]. In the presence of K^+^, G-quartet RNA is very stable, blocking RT progression at its 3′ edge and resulting in a truncated transcription product. Conversely, in the presence of Na^+^, G-quartet structures are destabilized, and the RT can proceed to the end of the RNA [4] . The RT elongation test on *Sod1* mRNA did not reveal any K^+^-dependent stop of the polymerase (Figure S1), demonstrating that *Sod1* mRNA is not able to form a G-quartet structure. Moreover, FMRP/*Sod1* interaction was not dependent on the presence of Mg^2+^, which is necessary to stabilize the “kissing complex” RNA structure (unpublished data) [2]. Taken together, these findings suggest that FMRP binds to *Sod1* mRNA via a novel sequence/structure. We continued the characterization of the FMRP/*Sod1* mRNA interaction by testing the ability of *Sod1* mRNA to compete for the binding of the FMRP/G-quartet RNA structure [4]. Indeed, 5 nM unlabeled *Sod1* mRNA competed very efficiently (65%) with the previously identified N19 FMRP binding site in a gel-shift assay, whereas a negative control, N8 RNA (corresponding to nt 1–654 of *FMR1* mRNA that does not contain the G-quartet), was not able to compete for the same interaction (Figure 1C). To precisely define the region of *Sod1* mRNA interacting with FMRP, we generated three different constructs from *Sod1* encompassing its full-length cDNA: its 5′ UTR and a portion of its coding region (*Sod1–5′* region), a central part of the coding region (*Sod1-mid* region), and a fragment overlapping the end of the coding region and the 3′ UTR (*Sod1–3′* region) (Figure 2A). RNA sequences corresponding to each fragment were produced and tested for their ability to interact with FMRP. Only the *Sod1–5′* region (spanning nt −70 to +148 of *Sod1* mRNA) competed with N19 binding to FMRP with the same affinity as the full-length *Sod1* mRNA (3 nM concentrations of both cold probes compete for 50% of FMRP/N19 binding) (Figure 2B). To identify the sequence of *Sod1* mRNA that is recognized and bound by FMRP, we employed a site boundary determination method [4]. In this experiment, the 3′- or 5′-end-labeled *Sod1–5′* RNA was treated by mild alkaline hydrolysis in order to generate a pool of smaller fragments. The RNA fragments retaining the capacity to bind FMRP were selected on immobilized glutathione-S-transferase (GST)-FMRP, as previously described [4]. Bound RNAs were analyzed by electrophoresis on a denaturing polyacrylamide gel (not shown). The border positions were at −30 and +34 for 3′- and 5′-end-labeled fragments, respectively. This technique allowed us to define a 64-base region spanning both sides of the *Sod1* AUG start codon that is protected by FMRP. We subcloned this sequence, and we synthesized its corresponding RNA, generating the *Sod1–64* RNA. This RNA was bound specifically by FMRP, because it was able to compete for the FMRP/*Sod1* full-length mRNA interaction (Figure 2C). Interestingly, the FMRP/*Sod1–64* interaction is competed for by the N19 G-quartet-containing RNA to the same extent (unpublished data).

To assess which portion of FMRP was able to interact with *Sod1* mRNA, we produced protein fragments of the different RNA-binding domains of FMRP (e.g., KH1, KH2, and RGG-box-containing C-terminal domains) as recombinant proteins in a bacterial system [14], and we used them in binding assays with the *Sod1–64* RNA. Interestingly, we observed that *Sod1–64* RNA interacts only with the C-terminal domain of FMRP encompassing the RGG box (Figure 2D) and was not able to interact with any of the KH domains, even at high protein concentrations (Figure 2D). As described previously, the same C-terminal domain was also able to bind the G-quartet RNA structure [3].

To assess whether *Sod1–64* RNA binds FMRP in the same ionic conditions as the full-length *Sod1* mRNA, we performed a binding assay in the presence of either K^+^ or Na^+^. As shown in Figure 2E, no differences were observed in the protein/RNA interaction under both conditions.

In addition, we tested the abilities of different Fragile X Mental Retardation Protein (FXR1P) isoforms to bind *Sod1*, as we have previously done for G-quartet RNA structures [15]. We used FXR1P Isoe (only expressed in muscle) and Isoa and Isod (highly expressed in brain but not in muscle) in a filter binding assay using *Sod1* as a probe*.* We observed that all FXR1P isoforms bind *Sod1* mRNA with lower affinity compared with that of FMRP. However, substantial differences exist among the three isoforms. Indeed, the affinity of Isoe for *Sod1* is quite high, because approximately 20 nM competitor RNA is able to displace 50% of *Sod1* mRNA from Isoe. Conversely, the affinities of Isoa and Isod are very low. These findings are shown in Figure S2.

### Structure of the *Sod1* mRNA Region Interacting with FMRP

**Figure 3:**
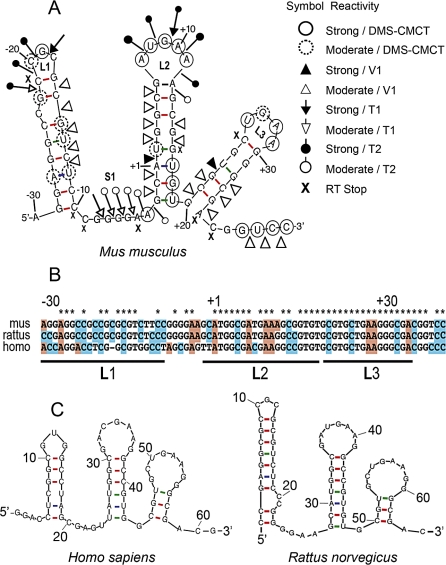
Secondary Structure of the SoSLIP RNA Fragment (A) RNA secondary structure model of the mouse *Sod1–64* RNA fragment (SoSLIP) showing results from enzymatic cleavage and chemical modification experiments. White and black arrows represent moderate and strong RNase T1 cleavage sites, respectively. White and black triangles represent moderate and strong RNase V1 cleavage sites, respectively. Symbols used to indicate the reactivity of different drugs or nucleases are shown in the figure; “X” represents RT pauses. (B) Alignment of SoSLIP sequence in mouse, rat, and human. (C) Conservation of the SoSLIP RNA secondary structure in rat and human.

To unravel the mechanism of action of the FMRP/*Sod1–64* interaction in translational control, we decided to determine *Sod1–64* RNA structure in the absence and in the presence of FMRP. To determine the secondary structure of *Sod1–64* RNA, we probed the structure of this 64-base region in solution, using a panel of chemical and enzymatic modifications, as described [16]. This technique is based on the reactivity of RNA molecules toward chemicals or enzymes that modify or cleave specific atomic positions in RNA. The probing experiments were performed using unlabeled or radioactively end-labeled in vitro transcribed RNAs (*Sod1–5′* region), which were subjected to random digestion with RNases T1, T2, and V1 or chemical modifications with dimethyl sulfate (DMS) and a carbodiimide derivative (CMCT). RNase T1 cuts after guanine residues in single-stranded regions, RNase T2 cleaves after all single-stranded residues, but preferentially after adenines, whereas RNase V1 cuts at double-stranded or stacked bases. DMS alkylates the N1 position of adenines and the N3 position of cytosines, whereas CMCT modifies the N1 position of guanines and the N3 position of uridines. The sites of cleavage or modification were then identified by primer extension with reverse transcriptase, using a radiolabeled primer complementary to the *Sod1*-5′ region. Analysis of the resulting cDNAs was performed on sequencing polyacrylamide gels that were run together with the corresponding RNA sequencing ladder to allow identification of the modified residues (Figure S3). A secondary structure model was further derived by combining experimental data and free energy data calculated using the mFOLD program (http://helix.nih.gov/apps/bioinfo/mfold.html). The structure of *Sod1–64* RNA appears as a succession of three independent stem-loop structures that are separated by short single-stranded regions (Figure 3A). *Sod1–64* appears strongly conserved at the sequence and structure level also in rat and human (Figure 3B and 3C). We called this FMRP-interacting structure SoSLIP.

**Figure 4:**
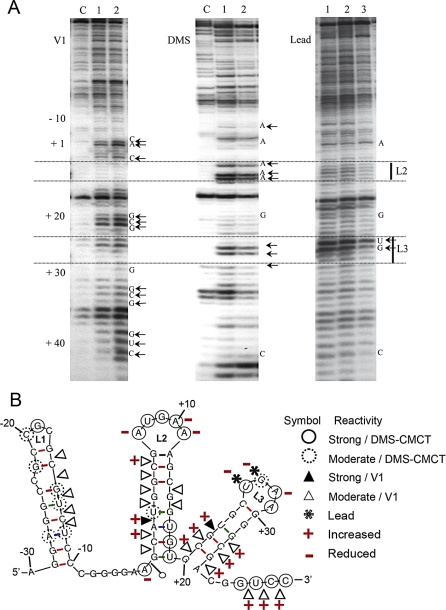
Chemical and Enzymatic Probing of the SoSLIP and Its Resulting Secondary Structure in the Presence and in the Absence of FMRP (A) PAGE gel showing the running of retrotranscribed SoSLIP RNA after treatment with RNase V1 (left), DMS (middle), and lead (right). “C” indicates the lane where SoSLIP was untreated, lane 1 is the treated RNA, and lanes 2 and 3 represent the SoSLIP RNA after incubation with an increasing amount of FMRP before being treated as described. The positions of nucleotides are indicated together with the region corresponding to the second (L2) and third (L3) loops. (B) RNA secondary structure model of SoSLIP showing results from enzymatic cleavage and chemical modification experiments in the presence of FMRP. The symbols indicating reactivity toward V1, DMS, or lead are shown on the right. The two symbols + and – were used to indicate an increased or decreased reactivity, respectively, upon the interaction with FMRP.

We then analyzed the SoSLIP structure by RNA protection after incubation with FMRP. SoSLIP RNA was treated by V1 nuclease, DMS, and Pb^2+^ in the absence or in the presence of increasing amounts of recombinant FMRP. The modified RNA was reverse-transcribed using the radiolabeled primer IV, and the obtained products were separated by PAGE (Figure 4A). Protections and increases of reactivity were found within a region extending from nucleotides −10 to + 34 (Figure 4B). Interestingly, lead, a probe specific for unpaired nucleotides, flexible regions, and protein binding sites, with no base specificity, revealed protections over this whole area. Because of their extent, these protections indicate a combination of both direct protection from the protein and strengthening of the structure. Noticeably, the hyperreactive positions +26 and +27 in L3 were particularly protected by the protein. A modification of the SoSLIP structure is further indicated by the increases of reactivity observed with V1 cleavages (for example, the (A) base of *Sod1* starting codon AUG and its preceding base (C), as well as the second stem and the more 3' part) (Figure 4B). In conclusion, these data indicate that FMRP protects the RNA particularly at the level of the L2 and L3 loops and induces conformational changes of the L2 and L3 stems, with the possible exposure of several nucleotides, and in particular the AUG sequence in the L2 stem, in the presence of the protein.

### Role of FMRP in Stability and Translatability of *Sod1* mRNA

**Figure 5:**
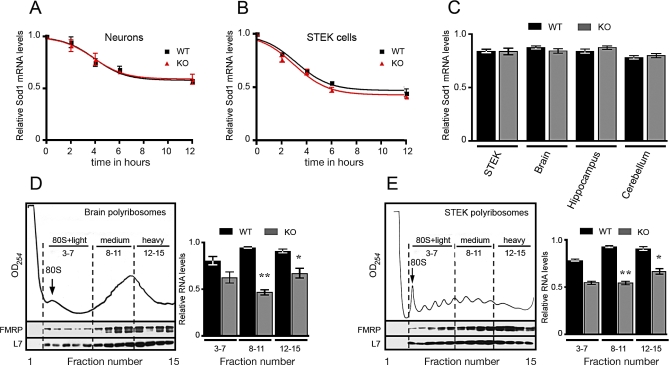
Stability and Translatability of *Sod1* mRNA (A) Primary cultured hippocampal neurons derived from *Fmr1* knockout or wild-type mice were incubated with 5 μM actinomycin D. Total RNA was extracted at different times (2, 4, 6, and 12 h) after the treatment, and *Sod1* mRNA was quantified by qRT-PCR as described [37]. All results are listed in Table S2. (B) STEK cells expressing or not expressing FMRP were incubated with 5 μM actinomycin D. Total RNA was extracted at different times (2, 4, 6, and 12 h) after the treatment, and *Sod1* mRNA was quantified by qRT-PCR as described [37]. Values are listed in Table S2. (C) Cytoplasmic RNA was extracted from cells and mice tissues expressing or not expressing FMRP. The *Sod1* mRNA level was normalized by the *Hprt* mRNA level by applying the formula: Ct *Sod1*/Ct *Hprt*. As shown in the diagram, the *Sod1* mRNA levels were not affected by the absence of FMRP, and no statistically significant differences were observed for *Sod1* mRNA levels in tissues and cell lines expressing or not expressing FMRP. Results are presented as the mean ± SEM. (D) Polyribosome association of *Sod1* mRNA in brain obtained from wild-type and *Fmr1* null mice. The UV profile of a sucrose density gradient is shown, and the 80S monosome peak is indicated. RNA purified from fractions corresponding to 80S and light-, medium-, and heavy-sedimenting polyribosomes were pooled, and the *Sod1* mRNA levels in each pool were determined by qRT-PCR by applying the formula: Ct *Sod1*/Ct *Hprt*. *Sod1* mRNA is less associated with medium and heavy polyribosomes in the absence of FMRP. Results are presented as the mean ± SEM (Student's *t*-test, **p < 0.01 for medium polyribosomes) (Student's *t*-test, *p < 0.05 for heavy polyribosomes). No statistically significant differences were observed for light polyribosomes. (E) Polyribosome association of *Sod1* mRNA in STEK cell lines expressing or not expressing *FMR1*. The UV profile of a sucrose density gradient is shown, and the 80S monosome peak is indicated. RNA purified from fractions corresponding to 80S and light-, medium-, and heavy-sedimenting polyribosomes were pooled, and the *Sod1* mRNA level in each pool was quantified as described in (D). *Sod1* mRNA is reduced in medium and heavy polyribosomes in the absence of FMRP. Results are presented as the mean ± SEM (Student's *t*-test, **p < 0.01 for medium polyribosomes) (Student's *t*-test, *p < 0.05 for heavy polyribosomes). No statistically significant differences were observed for light polyribosomes.

We investigated the impact that the absence of FMRP has on the stability and translatability of *Sod1* mRNA. To explore *Sod1* mRNA decay, we blocked transcription in primary cultured hippocampal neurons and in STEK cell lines by actinomycin D treatment. We did not observe any significant difference in *Sod1* mRNA levels of neurons obtained from wild-type or of *Fmr1* null mice (Figure 5A) or fibroblasts expressing or not expressing a *FMR1* transgene, even after 12 h of actinomycin D treatment (Figure 5B). Then, we analyzed the level of *Sod1* expression in cytoplasmic RNA extracts from STEK cells [5], total brain, hippocampus, and cerebellum of mice expressing or not expressing the *Fmr1* gene. With quantitative (q) RT-PCR, the amount of *Sod1* mRNA was found to be equivalent in both wild-type and *Fmr1* knockout cells and tissues when normalized to the level of *Hprt* mRNA (Figure 5C). All of these data excluded the possibility that FMRP would regulate the stability of *Sod1* mRNA. We then investigated the role of FMRP in *Sod1* mRNA translatability. FMRP being a well-known polyribosome-associated protein in brain [2,17–19] and all tissues and cell lines analyzed [20,21], we studied the distribution of *Sod1* mRNA in polyribosomes derived from extracts of STEK cells expressing or not expressing a *FMR1* transgene and from brain extracts of wild-type and *Fmr1* null mice. We used the polyribosome purification procedure previously described [19], because this method is based on the concentration of polyribosomal franctions, avoiding contamination of light mRNP. We evaluated the *Sod1* mRNA level by qRT-PCR using *Hprt* mRNA as an internal control. In the absence of FMRP, we observed a decreased level of *Sod1* mRNA in polyribosome fractions (medium and heavy) obtained from fibroblasts (Figure 5D), as well as in the corresponding polyribosomal fractions obtained from total brain (Figure 5E). Indeed, as we have shown in Figure 5D and 5E, the amount of *Sod1* mRNA associated with polyribosomes is dependent on the amount of FMRP, because a reduced association (statistically significant) is observed in medium and heavy fractions where the amount of FMRP is most abundant, and nonsignificant differences are observed in the light fractions where the amount of FMRP is less abundant. These results suggest that the absence of FMRP plays a key role in *Sod1* mRNA incorporation in the translating machinery.

### Sod1 Expression Is Impaired in *Fmr1* Null Mice

**Figure 6:**
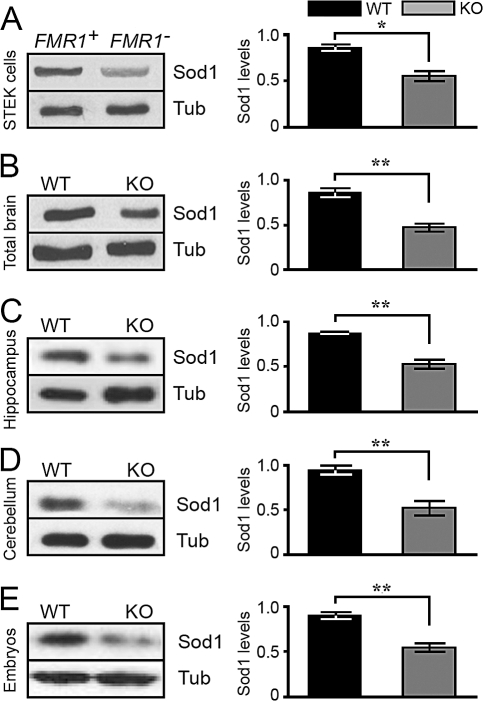
Decreased Levels of Sod1 Protein in *Fmr1* Null Cells, Brain, and Embryos Western blot analysis of one *FMR1* ^+^ STEK clone (where *FMR1* was reintroduced) and one STEK *FMR1* null clone. The results shown on the left are representative of the different clonal cell lines. On the right, corresponding densitometric analyses show a significant decrease of *Sod1* expression, after comparing five wild-type rescued clones and five *FMR1* knockout clones. Three independent experiments were quantified. Results presented as the mean ± SEM (Student's *t*-test, *p < 0.05) are the average of *Sod1* levels normalized for β-tubulin expression The same analysis described in (A) was applied for mouse total brain (B), mouse hippocampus (C), mouse cerebellum (B) and mouse 10dpc embryo extracts (E). Densitometric analysis showing a significant decrease in *Sod1* expression. Three independent experiments were quantified using eight wild-type and eight *Fmr1* null mice. Results presented as the mean ± SEM (Student's *t*-test, **p < 0.01) are the average of *Sod1* levels normalized for β-tubulin expression.

To assess whether the reduction of the association of *Sod1* mRNA with polyribosomes impairs the expression of the Sod1 protein in the absence of FMRP, we analyzed total protein extracts obtained from STEK cells expressing or not expressing a *FMR1* transgene [5], and we observed that Sod1 protein expression is reduced by approximately 40% in *Fmr1* null cells, as compared to that of cells expressing FMRP and after normalization of its expression to that of β-tubulin (Figure 6A). Similarly, we observed a significant decrease in Sod1 level in total protein extracts from whole brain (Figure 6B), hippocampus (Figure 6C), and cerebellum (Figure 6D) of 12-day-old *Fmr1* null mice, as compared to those of wild-type littermates. Sod1 levels were also reduced in *Fmr1* null mice embryos at 10 days post coitum (10dpc) (Figure 6E). We therefore concluded that Sod1 levels are directly correlated with the reduced association of its mRNA on medium- and heavy-sedimenting polyribosomes in *Fmr1* null mice, suggesting that FMRP promotes the association of *Sod1* mRNA to actively translating polyribosomes.

### Role of the FMRP/SoSLIP Complex in Translation

**Figure 7:**
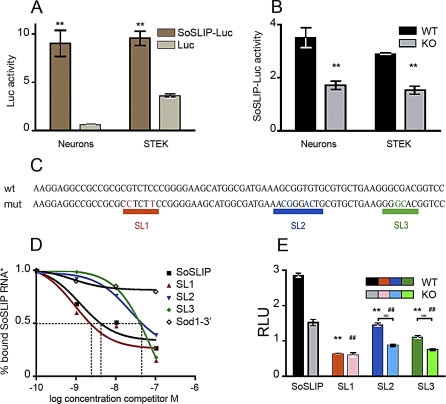
Impact of SoSLIP on Translational Regulation (A) Effect of SoSLIP sequence upon luciferase expression: luciferase activities of Luc or SoSLIP-Luc vectors in primary neurons and STEK cells. Three independent experiments with three replicates, done in triplicate, for each transfection were quantified. For each transfection, firefly(F) luciferase (luc) activity was normalized by *Renilla* (R) luciferase (luc) activity. Results are presented as the mean ± SEM (Student's *t*-test, **p < 0.01). (B) Activity of SoSLIP-Luc in neurons and STEK cells expressing or not expressing FMRP. Three independent experiments in triplicate for each transfection were quantified. For each transfection, Fluc activity was normalized by Rluc activity. Results presented here represent the mean ± SEM of the ratio of SoSLIP-Luc to Luc activities (Student's *t*-test, **p < 0.01). (C) Schematic representation of the wild-type SoSLIP sequence and its three mutants (SL1, SL2, and SL3). (D) Binding affinity of FMRP to wild-type SoSLIP and SL1, SL2, and SL3 mutants. Filter binding assay using radiolabeled SoSLIP and unlabeled cold RNA competitors SoSLIP, *Sod1–3′* region, SL1, SL2, and SL3. All of the results obtained in the filter binding assay are listed in Table S2. (E) Effect of SoSLIP mutants (SL1-Luc, SL2-Luc, and SL3-Luc) on luciferase expression in STEK cells expressing or not expressing FMRP. Three independent experiments in triplicate for each transfection were quantified. For each transfection, Fluc activity was normalized to Rluc activity. Results presented here represent the mean of the ratio of SoSLIP-Luc to Luc, SL1-Luc to Luc, SL2-Luc to Luc, and SL3-Luc to Luc. The luciferase activities of the three mutants were compared to wild-type SoSLIP luciferase activity in cells expressing FMRP, and the difference was significant in all cases (Student's *t*-test, **p < 0.01). The same analysis was repeated in cells not expressing FMRP, and the difference was significant in all cases (Student's *t*-test, ##*p* < 0.01). The luciferase activity of each mutant in cells expressing or not expressing FMRP was evaluated. For mutants SL2 and SL3, the reduction of lucferase activity observed in *Fmr1* null cells was statistically significant. These results are presented as the mean ± SEM (Student's *t*-test, °°*p* < 0.01). For mutant SL1, no significant reduction of luciferase activity was observed in cells not expressing FMRP compared with cells expressing FMRP. These results are presented as the mean ± SEM. RLU, relative luciferase units.

To confirm the positive role of FMRP in translational modulation of Sod1 expression by the interaction with the SoSLIP RNA structure, we cloned this sequence upstream of the luciferase gene in the pcDNA3.1 zeo vector (Luc) to evaluate the effect of the presence of SoSLIP on the expression of a reporter protein. We transfected primary cultured hippocampal neurons with the SoSLIP-luciferase vector (SoSLIP-Luc) or with the Luc vector, and we tested luciferase activity, showing on average an 8-fold increase when SoSLIP is placed upstream of the reporter (Figure 7A). A similar result was obtained in *FMR1*-expressing STEK cells (Figure 7A). Analysis of luciferase mRNA levels tested by qRT-PCR revealed that the presence of SoSLIP did not affect the mRNA expression level or stability of the dowstream reporter gene (Figure S4A and S4B). These results indicate that SoSLIP behaves per se as a translational activator in both cell types. We then transfected the same plasmid in primary hippocampal neurons obtained from normal and *Fmr1* null mice and in STEK cells expressing or not expressing the *FMR1* transgene. Indeed, in the absence of FMRP, luciferase activity resulted in a 2-fold reduction as compared with that in the presence of FMRP (wild-type condition) (Figure 7B). These data suggest that the presence of FMRP potentiates the ability of the SoSLIP sequence to positively modulate the expression of a downstream coding sequence independently of the cellular type. In addition, our findings are compatible with the notion that FMRP's roles in translation might be positive or negative as discussed [1,13]. To test the functional importance of SoSLIP stem loops, we disrupted each of the three stem loops by site-directed mutagenesis (Figure 7C). Using a filter binding assay, we then tested the ability of each mutant to compete for the binding of the FMRP/SoSLIP interaction. As shown in Figure 7D, the SL1 mutant is able to fully compete for SoSLIP binding (4 nM cold SL1 probe competes for 50% of wild-type SoSLIP), indicating that the disruption of SL1 does not affect FMRP/SoSLIP interaction. Conversely, the two SL2 and SL3 mutants poorly compete for SoSLIP binding to FMRP (60 nM concentrations of both cold probes compete for 50% of SoSLIP). The disruption of these two stem loops reduces their affinity for FMRP binding (Figure 7D) but did not abolish this binding, as suggested by comparing with the competition of an RNA sequence not bound by FMRP (Figure 7D, 3′ UTR *Sod1* RNA as the cold competitor). All three mutations affect the SoSLIP translational enhancer properties*,* reducing the level of luciferase activity (Figure 7E), if compared with the luciferase activity of the SoSLIP-Luc construct. Indeed, the activities of SL1-Luc, SL2-Luc, and SL3-Luc are reduced by 80%, 50%, and 60%, respectively, if compared with SoSLIP-Luc activity when these constructs have been transfected in cells expressing FMRP. The activities of SL1-Luc, SL2-Luc, and SL3-Luc are also reduced (62%, 44%, and 50%, respectively), if compared with SoSLIP-Luc activity when these constructs have been transfected in cells not expressing FMRP. Furthermore, in *Fmr1* knockout cells, the two SL2 and SL3 mutants have a reduced translational enhancing activity if compared with their activity in wild-type cells (Figure 7E) (40% and 33% reduced activity, respectively), confirming the data obtained by the in vitro binding. Surprisingly, the absence of FMRP does not modify the impact of the SL1 mutant on luciferase activity (Figure 7E), suggesting that also the SL1 integrity is necessary for the correct function of FMRP. To confirm that the effect of the mutants was only affecting translation efficiency, the expression and the stability of the mRNAs of all three SoSLIP mutants were tested, and no differences were observed with the wild-type mRNA, in the presence or in the absence of FMRP (Figure S4A–S4C).

These results suggest a complex translational regulation of *Sod1* mRNA via the SoSLIP structure.

### SoSLIP Acts as an IRES-like Element in an FMRP-Independent Manner

**Figure 8:**
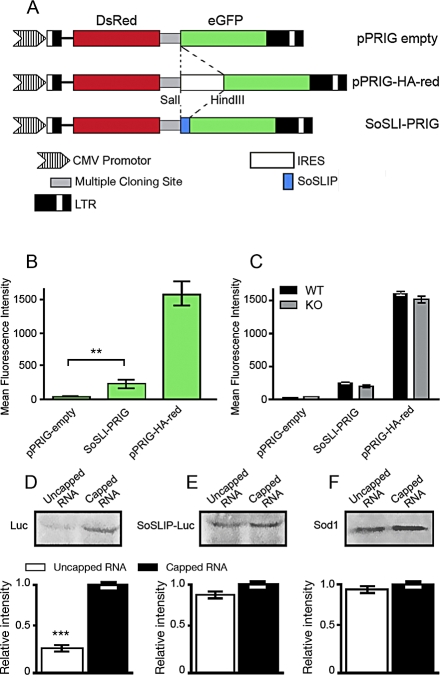
SoSLIP Acts as an FMRP-Independent IRES-like Element (A) Diagram of different constructs containing both DsRed and eGFP. These plasmids were modified by insertion of either a linker sequence (pPRIG-empty), the SoSLIP sequence (SoSLI-PRIG), or a characterized IRES (pPRIG-HA-red). (B) Histogram showing eGFP intensity (green) in a FACScan analysis on HeLa cells transfected with pPRIGempty, SoSLI-PRIG, or pPRIG-HA-red vectors. Two-hundred thousand cells positive for DsRed expression were analyzed for each transfection, and three independent experiments were quantified. The mean intensity of eGFP was calculated by the instrument software. Statistical analysis shows a significant difference between the mean intensity of GFP obtained by the pPRIGempty vector and that obtained by the SoSLI-PRIG vector (Student's *t*-test, **p < 0.01). (C) The same analysis described in (B) was repeated in STEK cells expressing or not expressing the *FMR1* transgene. Statistical analysis does not show a significant difference between the mean intensity of GFP in cells expressing or not expressing FMRP. Results are presented as the mean ± SEM. (D) In vitro translated capped and noncapped mRNA luciferase (Luc vector) in WGE. The relative intensity of each band was evaluated by densitometric analysis, and the values obtained are represented in the histograms. Four different experiments were quantified, and results are presented as the mean ± SEM (Student's *t*-test, ***p < 0.001). (E) The same experiment described in (D) was repeated for the in vitro translation of SoSLIP-Luc mRNA. Four different experiments were evaluated, and no statistically significant differences were observed. (F) The same experiment described in (D) was repeated for the in vitro translation of *Sod1* mRNA. Four different experiments were evaluated, and no statistically significant differences were observed. As in (D), in (E) and (F), results are presented as the mean ± SEM.

Due to its effects on translation, we asked then whether SoSLIP may act as an IRES. For this purpose, we used the pPRIG-HA-red bicistronic vector, where Discosoma sp. red fluorescent protein (DsRed) and enhanced green fluorescent protein (eGFP) are under the control of the same promoter. The DsRed is translated in a cap-dependent manner, whereas eGFP is translated only if an IRES sequence is cloned in front of it, as described [22]. We removed the IRES sequence (pPRIGempty), and we cloned the SoSLIP sequence between the DsRed and the eGFP cDNAs (SoSLI-PRIG) (Figure 8A). After 48 h of transfection, HeLa cells were analyzed by fluorescent-activated cell sorting (FACS), and the eGFP intensity was quantified for 200,000 cells expressing DsRed at a constant intensity. As shown in Figure 8B, we did not observe any green fluorescence in cells transfected with pPRIGempty, confirming that eGFP is not expressed in the absence of an upstream IRES sequence. Conversely, when SoSLIP is placed upstream of eGFP cDNA, green fluorescence becomes readily detectable. However, in this case, the mean eGFP fluorescence intensity was 5.4-fold less than that when using the strong viral IRES in pPRIG-HA (250 and 1,400 arbitrary units (AUs), respectively)*.* The same result was observed following transfection of the neuroblastoma NG108 cell line, neurons, COS cells, and STEK cells expressing *FMR1* (unpublished data). No differences were observed in the intensity level of GFP fluorescence from the SoSLI-PRIG vector in cells expressing or not expressing FMRP, suggesting that this protein is not required for the mild IRES-like activity of SoSLIP (Figure 8C). Even if FMRP cannot be considered as an IRES translational activating factor, the observation that SoSLIP may act as an IRES-like sequence is important to understand the role of SoSLIP in translational control. To confirm this result, we produced mRNAs encoding luciferase and SoSLIP-Luc carrying or not carrying the cap modification, and we translated in vitro equal amounts of each mRNA in rabbit reticulocyte lysate (RRL) and in wheat germ extract (WGE). As expected, luciferase mRNA is translated with higher efficiency when the mRNA is capped (Figure 8D). SoSLIP-Luc is translated in a cap-independent manner with efficiency comparable to that obtained in the cap-dependent manner (Figure 8E). The same result was obtained for the translation of *Sod1* mRNA in the capped and not capped versions (Figure 8F). No differences were observed using either the RRL or the WGE systems.

## Discussion

The primary function of FMRP resides in its ability to bind mRNAs. Despite the importance of this function, the RNA binding specificity of FMRP is not completely understood [23]. To date, only a single structure, the G-quartet, was found to mediate the specific interaction of FMRP with several of its target mRNAs [3–5,24]. A synthetic RNA with a specific structure, called the “kissing complex,” binds FMRP with high affinity but has not been found in any naturally occurring mRNA that is a target of FMRP [2,24]. The G-rich 3′ UTR of *PSD95* mRNA has been reported to interact with FMRP, and the authors claimed that this interaction happens via a novel motif [10]. The structure of this motif has not been defined, but a sequence highly similar (95%) to the G-quartet consensus previously determined [3] is present in the 3′ UTR of *PSD95*, strongly suggesting the presence of a G-quartet in *PSD95* mRNA [25]. Other mechanisms depending on the interaction of FMRP with noncoding mRNAs are controversial [26]. In conclusion, the specific sequence/region mediating the interaction of most putative mRNA targets with FMRP has not been experimentally defined. It is thought that elucidating the functional significance of the FMRP/RNA interaction is a critical step to understand the molecular bases of Fragile X syndrome. On the basis of conclusions from several laboratories, it has been considered that FMRP behaves exclusively as a translational repressor [4,27]. Recent studies have proposed a more complex function for FMRP, possibly depending on the specific binding of its target RNAs, on conformational changes in its structure, or on the influence of FMRP-interacting proteins [1,3,13,15]. Furthermore, a related member of the FXR family, FXR1P, was reported to function as a translational activator when associated with the AU-rich element present in the 3′ UTR of TNFα mRNA in response to serum starvation [28].

In this study, we define a new function of FMRP by dissecting the mechanism of binding of FMRP to the *Sod1* mRNA that was previously identified as an in vivo target of FMRP in cultured primary neurons [13]. Here we show that FMRP recognizes *Sod1* mRNA via a novel motif, the SoSLIP, organized in three stem loops separated by short sequences. In the absence of FMRP, *Sod1* mRNA present in polyribosomes is reduced, and Sod1 protein is less expressed in brains and cell lines from *FMR1* knockout mice, suggesting that Sod1 expression is positively modulated by the interaction between SoSLIP and FMRP. We have shown that the presence of FMRP protects the L2 and L3 loops of the SoSLIP structure. It is clear from our analyses that this interaction promotes structural modifications around the AUG start codon of *Sod1* mRNA and the more 3′ end portion of SoSLIP. These structural modifications apparently favor translation. SoSLIP is able to positively modulate the expression of a reporter gene, whose translation is also significantly increased by the presence of FMRP. We generated three mutants, each one impairing the formation of the three stem loops, respectively. All three mutants have a negative impact on the translational effect of SoSLIP. In addition, the absence of FMRP reduces the translational efficiency of the SL2 and SL3 mutants. These findings suggest that when SL2 is mutated the activities of SL1 and SL3 are probably still present, with SL3 activity being abolished in the *Fmr1* null cells. In a similar way, when SL3 is mutated, the activities of SL1 and SL2 are still observable in wild-type cells. These data are consistent with the in vitro binding results, showing an interaction of FMRP with the L2 and L3 loops, and with the finding that in both mutants the FMRP/SoSLIP interaction is reduced but not completely abolished. Interestingly enough, the SL1 mutation does not impair the binding of FMRP to SoSLIP but blocks its activity as a translational enhancer. We propose that the structural alteration due to the disruption of the SL1 stem prevents the conformational changes in SoSLIP structure upon interaction with FMRP that should promote translational activation. In this case, even if FMRP can recognize and bind SL2 and SL3 via the two loop structures, its function is abolished. Alternatively, FMRP needs the interaction with a factor(s) (probably binding to SL1) to carry out its function as an enhancer of translation. Due to the conformational changes of L2 and L3 stems induced by FMRP, we therefore propose that FMRP would facilitate ribosome scanning by participating in the remodelling of the SoSLIP structure, promoting in particular the exposure of the AUG of *Sod1* mRNA. This function is possibly cooperating with factor(s) binding the SL1 stem loop. Furthermore, our data suggest that the mechanism of action of FMRP is dependent on the type of RNA structure to which it binds. FMRP binds both G-quartet and SoSLIP RNAs through its C-terminal region containing the RGG box, even if in different ion concentrations. For this reason, it is tempting to speculate that in vivo the local ionic environment modulates the RNA binding properties for FMRP and favors the binding of either G-quartet or SoSLIP-containing mRNAs. This possibility might be particularly relevant in the synaptic compartment, because the binding of FMRP to its different mRNA targets might be directly modulated by depolarization.

In conclusion, SoSLIP can be considered as a “bipartite” translational activator: one domain (SL2 and SL3) acts in an FMRP-dependent-manner; the other (SL1) is independent of the presence of the Fragile X protein. Moreover, we also observed that SoSLIP may act as an IRES-like sequence. We observe that FMRP does not promote and/or influence this additional function of SoSLIP, suggesting that a specific mechanism of translational regulation is probably activated to translate SoSLIP-containing uncapped RNA. These data confirm the complexity of the translational regulation mediated by SoSLIP, and further studies will be necessary to fully understand its mechanism of action. Most important for our study, in this context, the function of FMRP is relevant to positively modulate SoSLIP-containing mRNA synergizing with other factor(s).

Sod1 is a well-known protein with antioxidant properties. Alterations of oxidative stress have been proposed to occur in *FMR1* null flies, because changes in the expression of proteins involved in redox reactions have been observed (1-cys peroxiredoxin in brain and peroxiredoxin and thioredoxin peroxidase in testis) [29,30], and a moderate increase of oxidative stress in the brain of *Fmr1* knockout mice has been recently described [11]. This modest impact of the absence of FMRP on brain oxidative stress might be due to the complex regulation of *Sod1* expression and the fact that the two FXR1P isoforms most expressed in the brain (Isoa and Isod) are also able to bind SoSLIP, even if with a lower affinity if compared with that of FMRP, suggesting that they can partially rescue FMRP function in *Fmr1* null cells. In addition, the FXR1P muscular isoform (Isoe) could functionally replace FMRP in muscle cells where this protein is absent. Moreover, modifications of oxidative stress have been linked to anxiety [31], sleep troubles [32], and autism [33], all phenotypic characteristics displayed by Fragile X patients [34]. Interestingly, chronic pharmacological treatment with α-tocopherol has been reported to reverse behavior and learning deficits of *Fmr1* knockout mice [35]. At the molecular level, Sod1 has been indicated as a regulator of growth factor signaling. In particular, Sod1 inhibition may attenuate phospho-extracellular signal-regulated kinase (pERK) signaling [12]. In this sense, it is remarkable that the rapid activation of ERK1/2 after metabotropic glutamate receptor 1 (mGluR1) stimulation is altered in *Fmr1* null mice [36], suggesting that reduced expression of Sod1 may contribute to this phenotype in *Fmr1* null synapses. In conclusion, our study suggests a role for Sod1 in the physiopathology of Fragile X syndrome and proposes a new function and novel mechanism of action for FMRP.

## Materials and Methods

### Plasmid constructs.

Primer sequences used to amplify *Sod1* and *FMR1* cDNAs are summarized in Table S1.

Mouse full-length *Sod1* (BC002066) and two of its deletion constructs (*Sod1-mid* region and *Sod1–64/*SoSLIP) were subcloned into the pGEM-T vector (Promega), and the *Sod1–3′* UTR construct was subcloned into the pCR2.1-TOPO vector (Invitrogen). SL1, SL2, and SL3 mutants were generated starting from the *Sod1–64* pGEM-T vector and using the QuickChange site-directed mutagenesis kit (Stratagene) and the oligonucleotides described in Table S1.

SoSLIP and its three mutants were cloned into the pcDNA3.1 zeo vector (Invitrogen) (Luc) using *Sod1–64*/SoSLIP HindIII primers described in Table S1, generating SoSLIP-Luc and SL1-Luc, SL2-Luc, and SL3-Luc constructs.

Sequences coding for KH1, KH2, and FMRP C-terminal domains were amplified by PCR from obtained *FMR1* ISO7 cDNA [14] using the appropriate primers. The PCR products were subcloned into the pET 151/DTOPO vector (Invitrogen), and the constructs were verified by sequencing.

For pPRIG-empty, the IRES sequence was removed by digestion of pPRIG-HA-red [22] with SalI and HindIII, filling in with the Klenow large fragment of DNA polymerase I and religation. SoSLI-PRIG was obtained by replacing the IRES SalI-HindIII fragment of pPRIG-HA-red with double-stranded oligonucleotides (Table S1) representing the sequence of SoSLIP. All constructs were verified by DNA sequencing.

### Immunoblot analysis.

Protein extraction and immunoblot analysis were performed as previously described [5]. The antibodies used in immunoblot analyses were used at the following concentrations: anti-FMRP antibody 1C3 1:10,000 [5], rabbit polyclonal anti-Sod1 antibody (Sod-100) (Stressgen) 1:5,000, monoclonal anti β-tubulin (E7) antibody (Iowa Hybridoma Bank) 1:5,000, and rabbit polyclonal anti-L7a antibody (a gift from A. Ziemiecki) 1:40,000.

### Protein expression.

Recombinant protein expression and purification were performed as previously described [15]. In vitro translated proteins (luciferase and Sod1) were produced using the RRL/WGE combination system (Promega). The mRNAs translated in these in vitro reactions were produced using T7 RNA polymerase (Promega) and using the mMessage mMachine kit (Applied Biosystems) specific for the synthesis of cap-modified mRNA. In both cases, we followed the manufacturer's protocol by starting from linearized plasmids (Luc, SoSLIP-Luc, and Sod1).

### RNA binding assay.

All RNAs were produced using T7 RNA polymerase (Promega), according to the manufacturer's protocol by starting from linearized plasmids. The pGEM-T and pTL1 vectors were linearized using PstI, and the pCR2.1 TOPO vector was linearized with BamH1. The *Sod1–5′* region was obtained by digesting pGEM-T *Sod1* full-length with BstXI. Restriction enzymes were purchased from New England Biolabs. RNAs were purified on NucAway spin columns (Ambion), and their qualities were verified on an polyacrylamide/urea gel after staining with Stains-All (Sigma). Protein/RNA interactions were analyzed either by electromobility shift assay or by filter binding assay, as previously described [4]. All of the experiments have been repeated at least three times. All values obtained are listed in Table S2.

### Structure-forming RNA detection.

The presence of a G-quartet structure in the *Sod1* mRNA was tested both by binding assay and by RT with different primers along the *Sod1* mRNA, as previously described, in the presence of Na^+^ or K^+^ in both experiments [4]. For the primer extension assays, RT was performed as described and using the following γ-^32^ATP 3′-end-labeled primers [4]:

Primer I 5′-CTCTTCAGATTACAGTTT-3′; primer II 5′-GTACGGCCAATGATGGAATG-3′; primer III 5′-GGATTAAAATGAGGTCCTGC-3′; primer IV 5′-CTTCTGCTCGAAGTGGATG-3′; primer V 5′-CTTCAGCACGCACGC-3′.

The *Sod1–64* RNA boundaries were determined as previously described [4].

### Cell cultures and transfections.

Primary cultures of hippocampal neurons were obtained from wild-type and *FMR1* null mouse embryos at 18 days of gestation. Primary cultures of cortical neurones were grown into 24-well format plates in 500 μl of Neurobasal Medium (Gibco) supplemented with 1× B27 (Gibco) and 0.5 mM l-glutamine in the presence of 100 IU/ml penicillin/streptomycin at 37 °C in 5% CO_2_. STEK *FMR1* null cells and its derivative stably transfected with *FMR1* cDNA [5] were grown as previously described [5]. Before transfection, cells were incubated in antibiotic-free medium. For the luciferase assay, transfections were performed in triplicate with Effectene (Invitrogen) as described by the manufacturer with 50 ng of the reporter gene (SoSLIP*-*firefly luciferase) and 5 ng of the plasmid coding for the *Renilla* luciferase used as a normalizer for each well.

### Luciferase assays.

Assays were performed 12 h after transfection. Starting from cell monolayers in 24-well cluster dishes, *Renilla* and firefly luciferase activities were determined using the Dual-Luciferase Reporter Assay System (Promega) and following the manufacturer's protocol. Luciferase activities were measured using a Luminoskan Ascent luminometer. AUs of luciferase activity were calculated accordingly the protocol of the luminometer manufacturer (Thermo Labsystem). In each transfection, firefly luciferase values were normalized with *Renilla* luciferase values.

### Chemical and enzymatic probing of the *Sod1–5*′ region to determine the SoSLIP structure.

The 5′ UTR of *Sod1* RNA (5 pmol) was renatured at 40 °C for 15 min in the appropriate native buffer (50 mM Hepes buffer pH 7.5 for DMS or borate buffer pH 8 for CMCT, 5 mM Mg Acetate, 50 mM KOH acetate, and 2 mM β-mercaptoethanol). Chemical modifications were performed in a final volume of 20 μl using either 1 μl of DMS diluted 1:2 (v/v) in ethanol or 60 μg of CMCT (carbodiimide) at 20 °C for 5 and 15 min, respectively, and in the presence of 2 μg of Escherichia coli tRNA. Enzymatic modifications were performed with V1 (0.0002 and 0.001 U), T1 (0.05 and 0.1 U), and T2 (0.05 and 0.1 U) nucleases, followed by a phenol/chloroform extraction. After ethanol precipitation and solubilization in the appropriate buffer, modified RNAs were reverse-transcribed using labeled primer III, and sequencing reactions and gel analysis were carried out as previously described [16]. In the presence of FMRP, experiments were performed using recombinant His-FMRP produced in a bacterial system [15] and GST-FMRP produced in a baculovirus system [4]. In the second case, protein was used attached to the glutathione beads treated with bovine serum albumin (1 μg/μl final concentration). RNA was incubated with the same amount of pretreated beads.

### RT-PCR.

The RT reactions were performed with 2 μg of RNA using the ThermoScript RT-PCR system (Invitrogen). All mRNAs extracted from transfected cells were treated with DNase I (4 U for each RT reaction) for 1 h at 37 °C before RT. DNase was removed by phenol/chloroform treatments, and the mRNA was recovered after ethanol precipitation. The PCR reactions were carried out with the qPCR Core kit for Syber Green I (Eurogentec) in an ABI PRISM 7000 instrument (Applied BioSystems). Primers used to amplify *Sod1* and the control *Hprt* are indicated in Table S1. Relative changes in mRNA amounts were calculated based on the 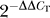 method [37].

### RNA stability.

STEK cells and neurons cultured in vitro for 10 days were treated with 5 μM actinomycin D (Sigma) for 2, 4, 6, and 12 h. Total RNA was purified from actinomycin-treated cells using the Qiagen RNeasy Mini Kit, and the RNA quality was verified on a 1% agarose gel and by optical density (OD) measurement. The *Sod1* mRNA level quantification was performed with the 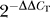 method [37].

### Polyribosome purification.

Polyribosome purification and analysis were performed following our previously described protocol [19] with minor modifications. Briefly, 1% Nonidet P-40 (NP-40) was added to the postmitochondrial supernatant, and 7 ml of the solution was layered over a 3-ml pad made of 45% sucrose in an 11-ml tube and centrifuged in a Sorvall TH-641 rotor at 34,000 rpm for 3 h. The ribosomal pellets were then resuspended in a buffer (20 mM Tris HCl, pH 7.4, 100 mM KCl, 1.25 mM MgCl_2_, 0.5% NP40, 1 U/ml RNAsin). Resuspended polyribosomes were analyzed by 15–45% sucrose gradients composed of 25 mM Tris HCl, pH 7.4, 100 mM KCl, 5 mM MgCl_2_, and 1 U/ml RNAsin. After centrifugation in a Sorvall TH-641 rotor for 2 h at 34,000 rpm and 4 °C, gradients were fractionated by upward displacement using an ISCO UA-5 flow-through spectrophotometer set at 254 nm and connected to a gradient collector. Fifteen fractions of 800 μl each were collected from the sucrose gradient. Approximately 100 μl of each fraction was ethanol-precipitated, resuspended in 50 μl of Laemmli buffer, and analyzed by immunoblot. The remaining 700 μl of each fraction was treated with Trizol (Invitrogen) to purify RNA. The quality of RNA was verified on a 1% agarose gel and by OD measurement. Three independent polyribosomal purifications were carried out for brain extracts and for STEK cell extracts.

### FACS analysis.

Cells were transfected for 48 h and after washing in phosphate-buffered saline were analyzed with a FACScalibur instrument (Becton Dickinson BD system). Cells expressing DsRed, with a constant intensity, were selected, and the mean of the eGFP intensity (expressed as AUs) of each sample was measured with the instrument software. The experiment was repeated three times in HeLa cells, COS cells, STEK cells expressing or not expressing FMRP, NG108 cells, and hippocampal primary neurons.

## Supporting Information

Figure S1 Primer Extension Assay Used To Detect the Presence of a G-Quartet Structure in the *Sod1* mRNA RT reactions were performed in the presence of 100 mM KCl or 100 mM NaCl, using the ^32^P- 5′-end-labeled primer I that hybridizes at positions +486; +464 (A), primer III (+194;+174) (B), or primer IV (+66;+46) (C). The resulting cDNA was separated on an 8% polyacrylamide/8 M urea sequencing gel and analyzed by autoradiography. RNA sequencing reactions were run in parallel. No specific RT stops were detected in the presence of potassium ion, thus excluding the presence of any G-quartet.
(1.5 MB TIF)

Figure S2 RNA Binding Properties of FMRP and FXR1P Isoforms (A) Filter binding assay using FMRP and FXR1P Isoe, Isod, and Isoa. The RNA probe used is ^32^P-labeled *Sod1* RNA, and competition was performed using the same unlabeled RNA.
(B) The same experiment was repeated using as a competitor the N8 RNA sequence, which we have previously shown [15] to be unable to bind either FMRP or FXR1P isoforms.
(4.2 MB TIF)

Figure S3 Enzymatic (A) and Chemical (B) Probing of In Vitro Transcribed *Sod1–5′* Region Cleavage and modification sites were detected by primer extension using the ^32^P- 5′-end-labeled primer IV. The resulting cDNA was separated on an 8% polyacrylamide/8 M urea sequencing gel and analyzed by autoradiography. RNA sequencing reactions were run in parallel. The nature and positions of different loops and stems are indicated at right. Increasing concentrations of RNase V1 (V1), RNase T1 (T1) (right), or chemical agents (DMS or CMCT) (left) were added before the reverse transcription step. The en-dash indicates the lanes where the untreated RNA was loaded.
(4.5 MB TIF)

Figure S4 Stability of SoSLIP-Luc and Its Mutants in the Presence or in the Absence of FMRP (A) Cytoplasmic RNA was extracted from STEK cells expressing or not expressing FMRP and transfected with Luc, SoSLIP-Luc, SL1-Luc, SL2-Luc, and SL3-Luc, respectively. Luciferase mRNA levels were analyzed by qRT-PCR and normalized to *Hprt* in each sample. As shown in the diagram, luciferase mRNA levels were not affected by the presence of SoSLIP or its mutants or by the presence or the absence of FMRP. No statistically significant differences have been observed for luciferase mRNA levels obtained from different constructs if compared with luciferase expressed from Luc. No statistically significant differences have been observed for luciferase mRNA levels in tissues and cell lines expressing or not expressing FMRP.
(B) STEK cells expressing FMRP were transfected with vectors Luc, SoSLIP-Luc, SL1-Luc, SL2-Luc, and SL3-Luc. Twelve hours after transfection, cells were incubated with 5 μM actinomycin D. *Sod1* mRNA levels were quantified at different times (2, 4, 6, and 12 h) after the treatment. Values obtained are shown in Table S2.
(C) *Fmr1* null STEK cells were transfected with vectors Luc, SoSLIP-Luc, SL1-Luc, SL2-Luc, and SL3-Luc. Twelve hours after transfection, cells were incubated with 5 μM actinomycin D. Total RNA was extracted, and *Sod1* mRNA levels were quantified at different times (2, 4, 6, and 12 h) after the treatment. Values obtained are shown in Table S2.
(6.2 MB TIF)

Table S1 Primer Sequences (27 KB PDF)

Table S2 List of Values Obtained in Experiments Described in Figures 1, 2, 5, 7, and Figure S2
(37 KB PDF)

## Abbreviations

*FMR1*: *Fragile X Mental Retardation 1*
FMRP: Fragile X Mental Retardation Protein
IRES: internal ribosome binding site
*Sod1*: *Superoxide Dismutase 1*
*SoSLIP*: mRNA Stem Loops Interacting with FMRP
