# Supplementary material for: A Novel Function for Fragile X Mental Retardation Protein in Translational Activation
Source: PLoS Biol. 2009 Jan 20;7(1):e1000016. doi: 10.1371/journal.pbio.1000016 (PMC2628407; doi:10.1371/journal.pbio.1000016)
Supplement: Supplementary file 1 [file pbio.1000016.st001.doc]

|  | | Forward | Reverse |
| --- | --- | --- | --- |
| *Sod1* full-length | | 5’CCCACGCGTCCGCTCGTC3’ | 5’CTCTTCAGATTACAGTTT3’ |
| *Sod1* mid-region | | 5’GGGTTCCACGTCCATCAGT3’ | 5’GGCCAATGATGGAATGCTC3’ |
| *Sod1* 3’UTR | | 5’CCGTACAATGGTGGTCCAT3’ | 5’CTCTTCAGATTACAGTTTAAT3’ |
| *Sod1*-64 | | 5’CGCCGCGCGTCTTCCG3’ | 5’GACCGTCGCCCTTCAGC3’ |
| FMRP-KH1 | | 5’CACCGCTAGTAAGCAGCTGG3’ | 5’CTAAAATTCGAGAAAGCTTCTAG3’ |
| FMRP-KH2 | | 5’CACCGCTGAAGATGTAATACAAGTTC3’ | 5’CTATAAATAGTTCAGGTGATAATCCAA3' |
| FMRP-FCT | | 5’CACCGCTCCAACAGAGGAAGAGAG3’ | 5’CTAGGGTACTCCAATCACGAGTG3’ |
| Sod1  q RT-PCR | | 5’ACCATCCACTTCGAGCAGAA3’ | 5’AGTCACATTGCCCAGGTCTC3’ |
| Hprt  q RT-PCR | | 5’GTAATGATCAGTCAACGGGGGAC3’ | 5’CCAGCAAGCTTGCAACCTTAACCA3’ |
| SL1 | | 5’GAGGAGGCCGCCGCGCCTCTTCCGGGG3’ | 5’CCCCGGAAGAGGCGCGGCGGCCTCCTC3’ |
| SL2 | | 5’GAAGCATGGCGATGAAACGGGACTGCGTGCTGAAGGC3’ | 5’GCCTTCAGCACGCAGCCCGTTTCATCGCCATGCTTC3’ |
| SL3 | | 5’CGTGCTGAAGGGGCACGGTCCGGTGC3’ | 5’GCACCGGACCGTGCCCCTTCAGCACG3’ |
| SoSLIP/Sal1  HindIII | | 5’GTCGACAAGGAGGCCGCCGCGCGTCTCCCGGGGAAGCATGGCGATGAAAGCGGTGTGCGTGCTGAAGGGCGACGGTAAGCTTGC3’ | 5’GCAAGCTTACCGTCGCCCTTCAGCACGCACACCGCTTTCATCGCCATGCTTCCCCGGGAGACGCGCGGCGGCCTCCTTGTCGAC3’ |
| Sod1-64 HindIII | 5’GGAAGCTTGGAGGCCGCCGCGCGT3’ | | 5’GGAAGTCCACCGTCGCCTTCAGCACGCAC  3’ |
| Luc1 FirFly | 5’GAATCCATCTTGCTCCAACAC3’ | | 5’TTCGTCCACAAACACAACTC3’ |
| Luc2 Renilla | 5’ATCAGGCAAATCTGGTAATGG3’ | | 5’ACATCTACTACACTTTCAGCGT3’ |

Table I
